# Scientific Items

**Published:** 1884-08-20

**Authors:** 


### SCIENTIFIC ITEMS.

**The Frontiers of Madness.**—This is the title of an interesting lecture recently delivered by Dr. Ball, in his course at the Paris Faculty of Medicine. The generally received opinion that folly and reason are separated by a strictly-drawn mathematical line is, according to Dr. Ball, quite erroneous. There is a broad frontier, he says, between sanity and insanity, which is peopled by millions of inhabitants. Damasippus, in Horace, laid down the doctrine that all men are mad—"*insanus et tu, stultique prope omnes.*" Dr. Ball, without going quite so far as this, holds that the number of persons perfectly reasonable on all points throughout the entire period of their existence forms but a minority of mankind. The world abounds with people, he tells us, whom a strict scientific diagnosis would condemn as mad, or more or less "touched;" yet, at no time of their life would it be permissible to put them under restraint. Such persons are to be seen occupying honorably and successfully every position in life and society. We brush against them when we take our daily walks abroad—we see them in the mirror which reflects ourselves.

Dr. Ball makes a classification of these "sane madmen," and assigns the first place to those who suffer from unreasonable, and, in most cases, irresistible impulses. He refers to the case of Dr. Johnson, and the curious impulse which prompted him to touch each post as he walked along the streets—an impulse so strong that if he accidentally passed one by without the usual tribute of a touch he felt irresistibly compelled to return and repair the omission. The overpowering impulse to laugh on occasions of peculiar solemnity is one which even the most serious persons have experienced. A still more morbid impulse is that which sometimes urges pious people to indulge in blasphemous or profane language. A great English divine, Bishop Butler, was tormented all his life long by this temptation, wbich he only mastered by strong and sustained efforts of the will. The impulse sometimes assumes a suicidal form.—*Pop. Science News.*

**Paraffine Paper.**—The paraffine or wax paper which is used in wrapping up candy, butter, cheese, provisions, tobacco, soap, druggists' supplies, kid gloves, hardware and other articles which it is wished to keep air and moisture tight is meeting with a largely increased demand every year, says an exchange. It is made by running tissue paper in the web, by means of rolling, through a bath of hot paraffine, with which it becomes thoroughly saturated. After leaving the bath, the paper passes through other rolls, which even the paraffine. It is then cooled by fans which "sets" the paraffine, and afterward wound into rolls. In 1877 14,500 reams of this paper were produced; in 1878, 28,000, and in 1879, 58,000 reams. In 1883 the product had reached 300,000 reams, and this year it is estimated 350,000 reams will be manufactured. The paper sells at $1 per ream. It was patented in

1877 by New York parties, and has been frequently infringed, but the courts have uniformly sustained the patentees, when the matter has been brought before them in suits at law.—*Nat. Drug.*

**Loganin.**—Messrs. Dunstan and Short claim that they have discovered the fruit of Strychnos nux vomica to contain from four to five per cent. of a new glucoside. They have named this loganin, and hope to soon be able to give the chemical, therapeutical and physiological properties of this constituent.—*Nat. Drug.*

**Dentistry in the United States.**—There are now about seventeen thousand dentists in the United States, and they pack into the teeth of the American people about a ton of pure gold and five times that amount of less precious metals (tin, silver, platinum, etc.) annually. Now, these metals are worth a million dollars, and it will take only about three hundred and fifty years to bury all the coin in the United States in the graveyards (another feather in favor of cremation).

There are about four millions of artificial teeth made in the United States yearly, yet only one-third of the people avail themselves of this blessing.

*Perfect* teeth are to be found in the mouths of only one American in eighty, the dental organs of seventy-nine being more or less affected.

This state of affairs will never improve until mothers are taught to bear children with perfect teeth and preserve them intact until the offspring is twenty years of age.—*Scientific Californian.*

**Water-glass Polish for Floors.**—A beautiful, lasting and fire-proof polish or covering for floors is made, according to the Wiener Gewerbeblatt, from water-glass. The floor is first cleaned and all cracks filled with a putty made of powdered glass in water-glass. After this is done water-glass of the consistence of paint or syrup is applied with a stiff brush. When the first coat is dry, a second, consisting of water-glass in which any mineral pigment has been stirred, is applied. Vegetable pigments will not do. To give a finer polish and make it more enduring, a third or even fourth coating of water-glass may be given.—*Nat. Drug.*

**Dilute Solution of Corrosive Sublimate.**—Sir Joseph Lister, in the British Medical Journal, has proposed the following method of making a solution of one part corrosive sublimate to one thousand menstruum. Make a solution of one part by weight of corrosive sublimate in one and one-half parts of glycerine; this will contain two-fifths of its weight of the sublimate. One drachm of this in four pints of water gives a solution one to one thousand. By keeping the solution in glycerine as a stock solution, a liquid, either alcoholic or aqueous, of any desired strength can readily be made.—*Nat. Drug.*

**Bringing up Children.**—Who knows how to bring up a child in the way he should go, so that when he's old he'll not depart from it?

